# Evaluation of Nanostructured Lipid Carriers Produced with Interesterified Buriti Oil

**DOI:** 10.17113/ftb.58.03.20.6195

**Published:** 2020-09

**Authors:** Lívia Viana de Castro Reis, Karina Magna Leão, Paula Speranza, Ana Paula Badan Ribeiro, Gabriela Alves Macedo, Juliana Alves Macedo

**Affiliations:** 1 Faculty of Food Engineering, Department of Food and Nutrition, State University of Campinas, Monteiro Lobato St. 80, Campinas, SP 13083-970, Brazil; 2 Faculty of Food Engineering, Department of Food Technology, State University of Campinas, Monteiro Lobato St. 80, Campinas, SP 13083-970, Brazil

**Keywords:** buriti oil, enzymatic interesterification, structured lipids, nanocarrier, droplet size, antioxidant activity

## Abstract

**Research background:**

Extracted from the pulp of an Amazonian fruit, buriti oil is rich in micronutrients with antioxidant properties and high biological value. The few studies available indicate that this oil could be used in a wide range of applications; however, there are no studies that work on the improvement in the characteristics of this oil for commercial application. The enzymatic interesterification is one of the tools available to improve the properties of oils and fats and our recent studies have demonstrated that the lipase could specifically act on buriti oil to produce structured lipids rich in oleic acid, while preserving most of the minor compounds present in this oil. Still looking for ways to expand the applicability of this raw oil, in this work, we are interested in studying the behaviour of this structured oil in nanostructured lipid carriers (NLCs).

**Experimental approach:**

The NLCs were produced with interesterified buriti oil and the stability, droplet size, electrical charge, microstructure, polymorphism and antioxidant activity of the samples were evaluated by ORAC and FRAP methods.

**Results and conclusions:**

The results showed that the interesterification formed more unsaturated triacylglycerols (TAGs), and NLCs prepared with interesterified buriti oil had smaller droplets than NLCs with crude buriti oil. Particles remained stable throughout the storage period and NLCs exhibited complex polymorphism with the presence of three crystalline forms. The oxygen radical absorbance capacity (ORAC) value was approx. 23% higher in nanolipid carries with structured lipids than in the nanolipid carriers with crude buriti oil, and the ferric reducing antioxidant power (FRAP) value 16% higher, demonstrating the influence of interesterification on the antioxidant activity of nanocarriers. Thus, NLCs prepared with interesterified buriti oil had small droplets, high stability and antioxidant capacity, and have a potential for nutritional and biological applications.

**Novelty and scientific contribution:**

This research showed that interesterification positively influenced the physicochemical properties of NLCs, producing the oil rich in oleic acid, high stability and antioxidant capacity. Therefore, it may be interesting to use these nanocarriers to obtain efficient carrier systems for future applications.

## INTRODUCTION

The Amazon region has suitable climatic conditions for a large number of underexploited native and exotic palm trees with interest to the agricultural industry, which can offer a future income source for local people. Buriti palm tree is an example that has high ecological, cultural and economic value, mainly due to its fruits that have valuable oil for the industries. It is estimated that the average annual production of pulp is approx. 0.79 t/ha and of oil 17.0 kg/ha (*1*). Extraction by cold pressing yields about 45 kg of buriti oil from 1000 kg of ripe fruits, which is considered as valuable resource for cosmetic, food, polymeric and pharmaceutical industry (*2*, *3*). It is commonly used by the local population as a healing agent, sunscreen, or for the treatment of burns, prevention of skin ageing, and even as anti-inflammatory and antibiotic agent (*3*). Buriti oil is rich in micronutrients with antioxidant properties andhas high biological value.It contains a high concentration of monounsaturated fatty acids and their content is very similar to olive oil; oleic acid is the major component of buriti oil, followed by palmitic acid (*4*). In addition, the oil is rich in minor compounds, such as carotenoids, tocopherols and sterols (*5*). Buriti oil is also a source of phenolic compounds (*3*), which are present in concentrationsgreater than those commonly found in other vegetable oils.

However, there are no studies that evaluate the improvement in the characteristics of these oils in order to expand their application. The enzymatic interesterification is one of the processes available to improve the properties of these oils and fats. Recent studies from our research group have shown that enzymatic interesterification can improve the biological characteristics of buriti oil, expanding its potential for applications in the development of cosmetic, pharmaceutical and food products with functional and medicinal effects. Speranza *et al.* (*6*) subjected the Amazonian buriti oil and murumuru fat to enzymatic interesterification using two lipases in three different enzyme systems. The three enzyme systems were able to catalyze the reaction, but the enzymes showed different specificities, producing interesterified lipids with different properties. The same research group (*7*) evaluated the antimicrobial potential of emulsions formulated with interesterified Amazonian oils. The results suggested that the interesterification of these oils may be responsible for changes in the physicochemical characteristics of the emulsions, producing droplets with smaller size and greater antimicrobial activity.

In this work, we are interested in studying the behaviour of this structured buriti oil in nanostructured lipid carriers. In recent years, several companies have developed foods and beverages with nutraceutical and functional properties based on the use of special oils, stimulating the emergence of novelcarrier systems for lipids with active compounds. For instance, nanostructured lipid carriers (NLCs) have been successfully applied in several areas to improve the solubility, bioavailability and stability of active compounds. NLCs might be an appropriate alternative to deliver structured buriti oil in foods and cosmetics as a functional ingredient and as vehicles or delivery systems for other lipophilic compounds, such as drugs, nutraceuticals, flavourings, antioxidants and antimicrobial agents.

NLCs are composed ofa mixture of solid and liquid lipids. The purpose of these formulations is to produce particles in which the oil phase is incorporated into the core of a solid lipid phase. They have high carrying capacity and provide controlled release of the active compounds (*8*). Thus, NLCs can enhance encapsulation efficiency, active compound binding and physical stability, and may be a valuable option to improve the chemical stability, bioavailability and controlled release of lipophilic compounds in functional foods. NLCsare able to immobilize active compoundswithin the solid lipid matrix, protecting them from degradation (*9*) by acting as a physical barrier against aqueous phase components.

For all that, weaimedto study the viability of producing NLCs with the structured buriti oil, and the characteristics of these nanoparticles formed with this special oil for future application as delivery systems in food and pharmaceutical industries.

## MATERIALS AND METHODS

### Materials

Crude buriti oil was purchased from Beraca Sabará (São Paulo, SP, Brazil). Compritol 888 ATO (glyceryl behenate, a mixture of mono-, di- and triacylglycerols from behenic acid) was bought from Brasquim (Porto Alegre, RS, Brazil). Immobilized *Thermomyces lanuginosus* lipase (Lipozyme TL-IM) was obtained from Novozymes Latin America Ltda (Araucária, PR, Brazil). All other reagents and solvents were of analytical grade.

### Enzymatic interesterification

Enzymatic interesterification of buriti oil was performed in an orbital shaking water bath (model TE-0532; Tecnal, Piracicaba, SP, Brazil), for 6 or 24 h at 150 rpm and 40 °C using 10 g of buriti oil and 2.5% (*m/m*) Lipozyme TL-IM (*6*). After the reaction was completed, the interesterified oil was immediately filtered through a 0.45-μm membrane. The filtrate was purified with 96% ethanol at 40 °C to remove free fatty acids. A stream of pure nitrogen was passed through the reaction mixture to prevent fat oxidationand samples were stored in a freezer at −18 °C.

### Structured oil characterization

#### Fatty acid composition of buriti oil

The fatty acid composition of buriti oil was determined by gas chromatography (model GCMS-QP2010S; Shimadzu, Tokyo, Japan), coupled to an FID flame ionization detector according to AOCS method Ce 1-62 (*10*). Fatty acid methyl esters were separated according to Hartman and Lago's method (*11*) on Agilent DB–23 (Santa Clara, CA, USA) capillary column ((50% cyanopropyl)-methylpolysiloxane), *l*=60m, *d*_int_=0.25 mm, *δ*_film_=0.25 μm. The following oven temperature schedule was employed: 110 °C for 5min, 110-215 °C (5 °C/min), 215 °C for 24 min; detector temperature 280 °C; injector temperature 250 °C, column flow 1.0 mL/min, linear velocity 24 cm/s, entrainment gas helium, 1:50 split, injected volume 1.0 μL. The molar fatty acid composition was calculated using the reference standard for the mixture of fatty acid methyl esters (FAME) obtained from Sigma-Aldrich, Merck (Supelco 37 Component FAME mix, Darmstadt, Germany). FAME were quantified using relative peak areas. Analyses were carried out in duplicate, and the mean and standard deviations of each sample were calculated (*12*).

#### Lipid class analysis

Lipidclass (triacylglycerols (TAG), diacylglycerols (DAG), monoacylglycerols (MAG), and free fatty acids) analysis of buriti oil and structured buriti oil was performed byhigh-performance sizeexclusion chromatography (HPSEC). The samples were diluted to 1:100 in tetrahydrofuran and analyzed in a liquid chromatograph equipped with a Rheodyne 7725i injector and a Waters 510 pump (Waters Associates, Milford, MA, USA) with two Ultrastyragel columns (100 and 500 Å; Waters Associates), each with 25 cm×0.77 cm i.d., packed with a styrene–divinylbenzene copolymer (*d*_internal_=10 mm), connected in series, and a refractive index detector (Hewlett Packard, Palo Alto, CA, USA). Conditions were the following: mobile phase tetrahydrofuran (HPLC grade), flow 1 mL/min, and injection volume 20.0 µL (*13*). The classes of compounds were identified by comparing the elution times with triacylglycerols, diacylglycerols, monoacylglycerols and free fatty acid standards and the analysis was performed in duplicate.

#### Triacylglycerol composition

The TAG composition of buriti oil was analyzed by reversed-phase HPLC, following the method described by Holčapek *et al.* (*14*) and adapted by Carvalho *et al.* (*15*). The chromatographic peaks of the samples were compared with the retention times of chromatographic peaks of soybean oil, which was used as a reference sample (*16*) and identified based on the expected elution order (considering that retention is increased), and on the fatty acid composition of the samples that were used as a reference. TAG composition of the samples was determined by internal normalization. Analysis was carried out in duplicate.

#### Determination of tocopherols

The levels of α-, β-, γ- and δ-tocopherols were determined according to AOCS method Ce 8-89 (*17*). Samples were diluted in hexane at a concentration of 0.1 mg/mL and then injected in UHPLC Ultimate® 3000 liquid chromatograph (Dionex, Sunnyvale, CA, USA) with a PerkinElmer (Waltham, MA, USA) Series 200a fluorescence detector (290 nm excitation and 330 nm emission).The microparticulate silica column *l*=250 mm, *d*_int_=4 mm, with each particle measuring approx. 5 μ with mobile phase composed of HPLC grade hexane (99%) and isopropanol (1%) was used.

The qualitative composition of tocopherols was determined by comparison of peak retention times with those of tocopherol standards (α-, β-, γ- and δ-tocopherols), and the quantitative composition was determined by normalizing the area under the curve. Analysis was performed in triplicate, and mass fraction of tocopherol in oil samples was expressed in mg/kg. The conversion to α-tocopherol equivalent unit (α-TE) was obtained through coefficient 1 for α-tocopherol, 0.5 for β-tocopherol, 0.1 for γ-tocopherol and 0.03 for δ-tocopherol, according to Darnet *et al*. (*18*).

#### Determination of β-carotene

β-Carotene content was determined spectrophotometrically, according to the method of de França *et al.* (*19*). An aliquot of 0.1 g of oil was diluted in 25 mL of a 7:3. solvent mixture of *n*-hexane and acetone (>99%, Merck, Darmstadt, Germany) and absorbance was read at 453 nm using a UV-Vis Agilent (Agilent Technologies, Waldbronn, Germany) spectrophotometer. Carotenes in the extracts were calculated in terms of β-carotene, using a standard absorbance curve calibrated with β-carotene (>99%, Merck, Darmstadt, Germany). The standard curve was prepared with 0.1 g of β-carotene diluted up to 100 mL with the 7:3 solvent mixture of *n*-hexane and acetone. Aliquots were taken from this solution and diluted to five different concentrations. Absorbance was read at 453 nm. Results are given in μg of β-carotene per g of buriti oil. The analysis was performed in triplicate.

#### Phenolic compound determination

Phenolic compounds were determined by the Folin–Ciocalteu method, according to Hrncirik and Fritsche (*20*). This method is based on the reduction of phosphomolybdic and phosphotungstic acids by phenolic hydroxyls, which produces a blue colour. Phenolic compounds were extracted from the oil with a solution of water/methanol 60:40. Folin-Ciocalteu reagent (Sigma-Aldrich, Merck, St. Louis, MO, USA) was added to suitable aliquots of the extracts. After 3 min, a sodium carbonate solution (35%) was added to the mixture, which was diluted with water to a final volume of 1 mL. Absorbance was measured after 2 h at 725 nm on a Shimadzu spectrophotometer UV-1800 (Kyoto, Japan). The blank contained all the constituents of the reaction except phenolic solution, which was substituted by distilled water. A calibration curve was prepared using a standard solution of gallic acid (Sigma-Aldrich, Merck) at concentrations of 0.01–0.1 μg/mL. Analysis was performed in triplicate, and results are expressed in μg of gallic acid equivalents per g of buriti oil.

### Nanostructured lipid carrier preparation

NLCs were prepared according to the method of Müller *et al*. (*21*) and Averina *et al.* (*22*). NLC aqueous dispersions containing 10% (*m/m*) lipid phase were prepared by hot high-pressure homogenization. The lipid phase, composed of a 1:1 (*m/m*) mixture of solid lipids (Compritol 888 ATO) and buriti oil (non-interesterified, interesterified for 6 h, or interesterified for 24 h), was previously heated to 85 °C. The aqueous phase, composed of 1.2% Tween 80 emulsifier in deionized water, was also previously heated to 85 °C. Then, the lipid phase was added to the aqueous phase under continuous stirring using an Ultra-Turrax T25 (IKA, Staufen, Germany), and the mixture was kept at 85 °C and 7000 rpm for 5 min. The samples were subsequently homogenized in a high-pressure homogenizer (GEA-Niro-Soavi, Parma, Italy) at 85 °C and 8·10^4^ kPa for 3 cycles. The oil-in-water emulsion was cooled to room temperature to promotethe recrystallization of the lipid phase, and thus NLCs were formed (*23*). NLCs prepared with non-interesterified buriti oil (NLC_BO_), with buriti oil that was interesterified for 6 h (NLC_BO6h_), and with buriti oil that was interesterified for 24 h (NLC_BO24h_) were stored at refrigeration (4 °C) and room (25 °C) temperatures.

### NLC characterization

#### Droplet size measurement

The droplet size distribution of NLCs was measured using dynamic light scattering (Mastersizer 2000; Malvern Instruments, Malvern, UK) (*24*). Results are presented as Z-average particle diameters. The refractive indices of the dispersed and continuous phases used in these calculations were 1.46 and 1.33, respectively. Analyses were performed in triplicate with all samples: NLC_BO_, NLC_BO6h_ and NLC_BO24h;_ after 1, 15 and 30 days of storage at refrigeration (4 °C) and room (25 °C) temperatures.

#### Zetapotential measurement

Zetapotential (caused by electric charges around droplets) can provide information about interfacial properties. Zeta potential was determined using Zetasizer Nano-ZS equipment (Malvern Instruments) and samples were diluted (1:100) in distilled water (*25*). Analyses were performed in triplicate with NLC_BO_, NLC_BO6h_ and NLC_BO24h_ after 1, 15 and 30 days of storage at refrigeration (4 °C) and room (25 °C) temperatures.

#### Confocal laser scanning microscopy

Confocal laser scanning microscopy was carried out using a TCS SP5 II microscope (Leica Microsystems, Heidelberg, Germany). The oil phase of NLCs (100 μL) was dyed with10 μL of Nile Red solution in ethanol (1 mg/mL). Samples were mounted on glass slides and examined under 10× and 63×magnification at an excitation and emission wavelength of 543 and 605 nm, respectively. Autofluorescence was analyzed using the built-in software LAS lite (Zeiss Inc., Toronto, Canada) to evaluate the morphology of fat crystals (*26*). Analyses were performed with NLC_BO_, NLC_BO6h_ and NLC_BO24h_ after 1, 15 and 30 days of storage at room (25 °C) temperature.

#### Polymorphism analysis

The polymorphic forms of fat crystals were investigated by X-ray diffraction, according to AOCS method Cj 2-95 (*27*). Analyses were performed using a PW 1710 Philips diffractometer (Malvern Panalytical, Almelo, The Netherlands) with Bragg–Brentano geometry (θ:2θ) and Cu-Kα radiation (λ=1.54056 Å) at 40 kV and 30 mA. Step sizes of 0.02° (2θ scale) and an acquisition time of 2 s were used in the scanning range of 5–40° (2θ scale). Polymorphic forms were identified by the characteristic short spacings of each crystal. Analyses were performed with NLC_BO_ and NLC_BO24h_ after 30 and 120 days of storage at room (25 °C) temperature.

#### Antioxidant activity assay

Oxygen radical absorbance capacity (ORAC) assay was performed in a 96-well plate (TPP Techno Plastic Products AG, Trasadingen, Switzerland), using fluorescein (Sigma-Aldrich, Merck) as a fluorescent probe, according to the procedures of Dávalos *et al.* (*28*). The measurements were performed using a microplate reader (FLUOstar OPTIMA; BMG Labtech, Offenburg, Germany) with fluorescence filters at 485 nm excitation and 520 nm emission. The reaction, performed at 37 °C, was started by thermal decomposition of 2,2’-azobis(2-methylpropionamidine) (AAPH; Sigma-Aldrich, Merck, Steinheim, Germany) in a 75-mM phosphate buffer (pH=7.4). ORAC values were calculated using the difference between the area under the fluorescein decay curve of the sample and the blank (net area under the curve). Standard curves were constructed using 6-hydroxy-2,5,7,8-tetramethylchroman-2-carboxylic acid (Trolox, Sigma-Aldrich, Merck) at concentrations between 30 and 1500 μmol/mL. Regression equations between the net area under the curve and antioxidant concentration were calculated. Final values are expressed in μmol Trolox equivalents (TE) per mL of dry matter and analyses were performed in triplicate with NLC_BO_ and NLC_BO24h_.

#### Ferric-reducing antioxidant power assay

FRAP assay was carried out according to Benzie and Strain (*29*). In the dark environment, aliquots of 30 μL of standard or blank samples were added to 90 μL of distilled water and 900 μL of the FRAP reagent (2.25 mL of 0.3 M acetate buffer (Riedel-de Haen, Seelze, Germany), pH=3.6, 225 μL of 10 mM TPTZ (2,4,6-tripyridyl-*s*-triazine; Fluka Chemicals, Switzerland) in 40 mM HCl and 225 μL of 20 mM iron(III) chloride solution. The mixture was vortexed and 200-μL aliquots were transferred to a clear 96-well microplate. Absorbance was read using a microplate reader (FLUOstar OPTIMA; BMG Labtech) at 595 nm and 37 °C for 30 min of reaction. To achieve the standard curve, Trolox® solutions were prepared at concentrations between 15 and 1500 μmol/mL. Results are expressed in μmol Trolox equivalents (TE) per mL of dry matter and analyses were performed in triplicate with NLC_BO_ and NLC_BO24h_.

### Statistical analysis

Results are presented as meanvalue±standard deviation of replicates. Results were compared by analysis of variance (ANOVA) using Statistica v. 7.0 (*30*), and p<0.05 was considered to be significant difference between the results.

## RESULTS AND DISCUSSION

### Buriti oil characterization

Before the interesterification reaction, fatty acid composition of buriti oil was analyzed and the interesterification was evaluated through triacylglycerol composition and lipid classes. The complete characterization was published in our earlier report (*12*). Nevertheless, in this paper, we summarize the most relevant data for the present discussion.

Fatty acid composition analysis showed that the tested buriti oil is rich in oleic (C18:1) (74.2%) and palmitic acids (C16:0) (19.8%). The main types of triacylglycerols (TAG) in buriti oil were POO (38.9%) and OOO (32.7%). After interesterification, there was a reduction in the levels of POO (32.7%) and an increase in the OOO (36.8%). These modifications affect the functionality of the oil and reduce the melting range, altering lubrication properties, mechanical performance, structure and nutritional properties (*31*). Lipid class analysis showed that TAG structure was preserved after 24 h of interesterification. The 24-hour interesterification promoted a small increase (from 6.7 to 9.2%) in diacylglycerol, but not the formation of monoacylglycerols or free fatty acids.

Buriti oil contained four tocopherol isomers (α, β, γ and δ) and the β-isomer ((83.6±1.8) mg/kg) was predominant in this oil. After 24 h of interesterification, there was a significant loss ((60.4±0.9) mg/kg) of this isomer. In a study by Darnet *et al*. (*18*), the most abundant fraction was also β-tocopherol (57 mg/kg). The four tocopherol isomers were also detected in previous work of our research group (*3*), but at different levels. Differences in the mass fractions of tocopherol isomers may be caused by the refining conditions of the oil or by the soil and climate conditions under which buriti palm trees are cultivated. In contrast to the observed in relation to tocopherol content, carotenoids were not affected by interesterification, *i.e.* β-carotene mass faction in structured buriti oil did not differ from that of the crude oil.

The mass fraction of total phenolics in buriti oil ((292.3±6.8) µg/g) was close to those found in other vegetable oils. After interesterification, total phenolic concentration increased ((329.8±10.2) µg/g), which was not an expected result. We have previously suggested (*12*) that this result was a consequence of improved solubilization of structured lipids in the reaction medium. However, further investigations are needed at this point.

#### Droplet size

NLCs have been shown to be carriers with great ability to disperse bioactive compounds. The addition of solid lipids to emulsions improves stability and allows greater control of the release of bioactive molecules (*22*).

In this study, NLCs prepared with interesterified buriti oil had small droplets, especially NLC_BO24h_ sample, which had droplets that were 46% smaller than those of NLC_BO_ (Table 1). This is a very interesting result, under the same process conditions but with the structured oil, we obtained smaller nanocarriers. The TAG modifications from the biotransformation reaction may have influenced the particle size. The interesterification formed more unsaturated TAG (OOO), we believe, exerting a positive effect on NLC droplet size, even after the addition of Compritol 888 ATO to the system. Nanocarriers prepared with non-interesterified oils have larger droplets and are less stable than those prepared with interesterified oils, as their components have different properties (such as viscosity and melting temperature). Interesterification can produce single-phase lipids that do not undergo phase separation, which results in more stable emulsions (*32*). Furthermore, interesterification can reduce the types of TAGs initially present in the starting oil, thereby forming a lipid phase with a narrower range of polymorphic forms, greater capacity for interaction with other components, and more organized packing (*33*).

**Table 1:** Droplet size (nm) of nanostructured lipid carriers after 1, 15 and 30 days of storage at refrigeration (4 °C) and room (25 °C) temperatures

Temperature/°C	*d*_32_/nm			
*t*/day	NLC_BO_	NLC_BO6h_	NLC_BO24h_
4	1	(717.00±0.01)^b^	(524.00±0.01)^c^	(401.00±0.01)^d^
	15	(729.00±0.01)^ab^	(537.00±0.01)^c^	(393.00±0.01)^d^
	30	(729.00±0.03)^ab^	(538.00±0.01)^c^	(406.00±0.01)^d^
25	1	(748.00±0.01)^a^	(532.00±0.01)^c^	(393.00±0.01)^d^
	15	(737.00±0.01)^ab^	(530.00±0.01)^c^	(405.00±0.01)^d^
	30	(744.00±0.01)^a^	(529.00±0.01)^c^	(402.00±0.01)^d^

NLC_BO_=nanostructured lipid carrier prepared with non-interesterified buriti oil, NLC_BO6h_=nanostructured lipid carrier prepared with buriti oil interesterified for 6h and NLC_BO24h_ for 24 h. Results are expressed as mean value±standard deviation (S.D.); *N*=3. Values followed by different letters in superscript differ significantly (p≤0.05) according to Tukey’s test

Storage time and temperature did not influencedroplet size or stability. The properties of lipid nanoparticles are typically affected by many factors, including type and concentration of lipids and surfactants and viscosity of the lipid phase. It has been reported that particle size increases as the viscosity of the lipid phase increases (*34*) and that NLCs have smaller droplets when oil is added to the system, as liquid fats reduce lipid phase viscosity and surface tension. In contrast, increased surfactant concentration decreases particle size (*35*), because surfactants facilitate the fragmentation of nanoparticles during homogenization, reducing surface tension at the interface between solid and liquid phases.

In a study by Walker *et al.* (*36*), nanoemulsions were prepared using spontaneous emulsification with 10% total oil phase (50% fish oil and 50% lemon oil, by mass) and different surfactant/oil ratio. These nanoparticles were maintained at 5, 20 and 37 °C for 14 days to assess their physical stability. The surfactant concentration also had a large impact on the average particle diameter of the emulsions: as the surfactant/oil ratio increased, the mean particle diameter decreased. In addition, the mean particle size did not depend strongly on storage time or temperature, as occurred in our study. The fact that there was no significant change in the average particle size of the nanoemulsions after being stored at 5, 20 and 37 °C for 14 days suggests that they were stable against flocculation, coalescence and Ostwald ripening.

Yang *et al.* (*37*) investigated the effect of different liquid carrier oils on the crystallization and aggregation behaviour of tristearin NLC dispersions. The results demonstrated that NLC suspension stability was strongly affected by the type and amount of the carrier oil. Unsaturated fatty acids and short chain fatty acids result in lower melting points, affecting the stability of NLC dispersions. These results were confirmed by using olive oil (melting point of −6 °C) and palm oil (melting point of 35 °C) for the production of nanoparticles. Olive oil is rich in oleic acid, as is buriti oil; this property allowed for a much more stable dispersion than that obtained with palm oil.

#### Zeta potential of buriti oil droplets

Zeta potential is a parameter used to evaluate the stability and characteristics of particles. It indicates the electric charge on the surface of nanoparticles as well as the electrostatic repulsion between them. Table 2 shows the ζ-potential of NLCs prepared with buriti oil and structured buriti oil after 1, 15 and 30 days of storage at 4 or 25 °C.

**Table 2:** Zeta potential of nanostructured lipid carriers after 1, 15 and 30 days of storage at refrigeration (4 °C) and room (25 °C) temperatures

Temperature/°C			*ζ*-potential/mV	
*t*/day	NLC_BO_	NLC_BO6h_	NLC_BO24h_
4	1	(−26.1±0.7)^def^	(−24.8±0.6)^abcde^	(−23.1±0.6)^a^
	15	(−26.0±0.2)^cde^	(−25.6±0.3)^bcde^	(−23.9±0.1)^ab^
	30	(−26.2±0.5)^def^	(−26.0±0.2)^cde^	(−24.0±0.6)^abc^
25	1	(−28.1±1.0)^fg^	(−24.5±0.3)^abcd^	(−23.5±0.6)^a^
	15	(−26.7±1.3)^ef^	(−24.6±0.9)^abcd^	(−24.7±0.3)^abcd^
	30	(−24.8±0.6)^abcde^	(−29.3±0.9)^g^	(−24.6±0.9)^abcd^

NLC_BO_=nanostructured lipid carrier prepared with non-interesterified buriti oil, NLC_BO6h_=nanostructured lipid carrier prepared with buriti oil interesterified for 6 and NLC_BO24h_ for 24 h. Results are expressed as mean value±S.D. (*N*=3). Mean values followed by different letters differ significantly (p≤ 0.05) according to Tukey’s test

A minimum ζ-potential value of ±30 mV is required to prevent coalescence of droplets (*38*). Interesterification of buriti oil influenced the interfacial properties of droplets under the evaluated conditions. However, although NLC_BO_ initially had higher ζ-potential values than NLC_BO6h_ and NLC_BO24h_, differences among the ζ-potential values of NLCs became non-significant with storage time, indicating that interesterification helped maintain stability over time. The higher value of ζ-potential, positive or negative, the lower the probability of the droplets coalescing and better their stability (*39*). Thus, the physical stability of the nanostructured lipid carriers was maintained over time.

All dispersions exhibited negative ζ-potential values, showing that repulsion forces predominated over attraction forces. This property is desirable in NLCs, as it prevents the formation of aggregates and is an indicator of particle stability. NLCs, solid lipid nanoparticles and lipid emulsions also had a negative charge in a study by Fang *et al*. (*40*). The negative charge was caused by the anionic fractions of the lipophilic emulsifier (soybean phosphatidylcholine) and the fatty acid glycerides in the lipid core of Compritol and Precirol.

The stability of lipid nanoparticles is affected by the type and concentration of lipids. Niculae *et al.* (*23*) used different lipid matrices to prepare NLCs. Carnauba oil was added to the carrier system initially prepared with pomegranate seed oil only, and the ζ-potential increased from −21.7 to −30.9 mV. The components of carnauba oil might have disrupted the surfactant shell, causing a rearrangement of the surface charge and a consequent change in ζ-potential. In addition, the authors found that when oil concentration decreased, the physical stability of nanocarriers slightly increased. In a study by How *et al.* (*41*) three different formulations of NLC were prepared, containing olive oil/hydrogenated palm oil (HPO) ratios of 1:9, 2:8 and 3:7 and Polysorbate 80. These authors observed that increase in the oil to HPO ratio had caused increase in the ζ-potential of the NLC, while the increase in surfactant content decreased particle size.

Surfactants also play an important role in the stabilization of NLC lipid particles. Tween 80, the surfactant used in this study, is a non-ionic surfactant that provides steric repulsion through which the semi-solid particles of NLCs are stabilized. If the amount of surfactant is not sufficient, some lipid particles might be uncovered, which can lead to flocculation, aggregation and gelling (*42*). In a study by Niculae *et al.* (*43*), lipid nanoparticles prepared with Tween 80 as the main surfactant had better physical stability than those prepared with Tween 20, showing the importance of surfactants for nanoparticle stability. Thus, interesterification of buriti oil might have allowed for a better interaction between emulsifier and oil, providing greater stability.

#### Morphology of nanostructured lipid carriers

**Fig. 1:**
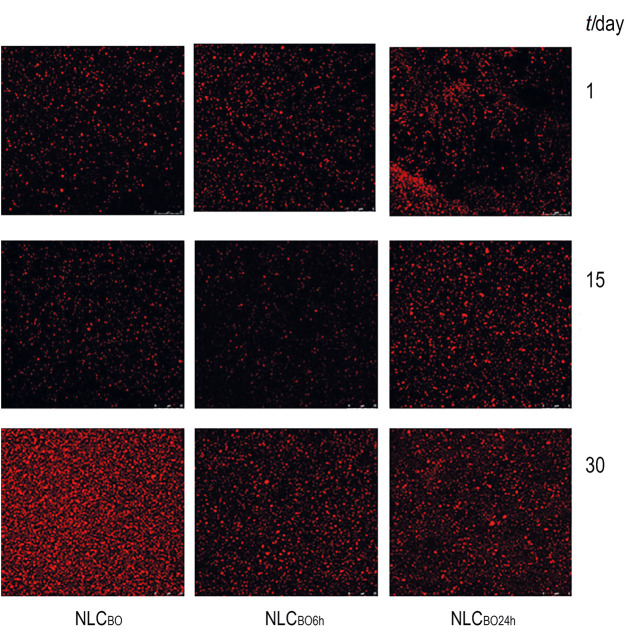
Influence of oil type on the morphology of nanostructured lipid carriers (NLCs). The oil phase is stained red, the scale bar is 25 μm. NLC_BO_=nanostructured lipid carrier prepared with non-interesterified buriti oil, NLC_BO6h_=nanostructured lipid carrier prepared with buriti oil interesterified for 6 and NLC_BO24h_ for 24 h

As storage temperature did not affect particle stability, microscopic analysis was not performed with samples stored under refrigeration. Fig. 1 shows the morphology of NLCs after 1, 15 and 30 days of storage at room temperature.

The confocal microscopy images (Fig. 1) show individual droplets with a spherical shape. In general, NLCs had a relatively wide droplet size range and droplets were polydisperse, without uniformity. The distribution of NLC_BO_ droplets was more homogeneous up to 15 days of storage, but droplets were more agglomerated after 30 days than those of other NLCs. NLC_BO6h_ showed a similar behaviour, but droplet aggregation was lower than that of NLC_BO_ after 30 days of storage.In contrast, NLC_BO24h_ had a more heterogeneous structure, exhibiting droplet aggregation throughout the storage period (*44*). Wegmüller *et al.* (*45*) reported the occurrence of particle agglomerates and fat crystals in the microcapsules developed in their study. Agglomeration might have occurred because microparticles were not completely solidified when cooled. The authors observed that small particles showed fewer fat crystals on the surface than larger ones, as small particles require less time to be totally solidified.

Different NLC morphologies are observed when using different microscopes, and these morphological discrepancies might be attributed to the type of lipid and surfactant or the method used to develop NLCs. Many studies suggest that incorporation of bioactive compounds into a solid lipid matrix can improve controlled release, charge, and physical and chemical stability by disrupting the crystal packing structure (*21*).

#### Polymorphism of nanostructured lipid carriers

TAGs occur in three crystalline forms, α, β′ and β, which differ in the arrangement of fatty acids. X-ray diffraction analysis was performed to investigate the arrangement of fatty acid chains and identify the polymorphism of crystals by determining the dimensions of the crystal unit and sub-cells. Polymorphs diffract X-rays at different angles as a result of their different geometric configurations, and a diffraction at wide angles corresponds to short spacings (distances between parallel acyl groups in TAG) of sub-cells, which allows the characterization of different polymorphs in fats (*46*).

The short spacings and polymorphic forms of NLC_BO_ and NLC_BO24h_ are presented in Table 3. NLCs show a complex polymorphism as a result of β′ fusion and solid lipid recrystallization. This behaviour can be explained by the nanosize of NLC particles, which cause slower transition to a stable state, and may also be related to the presence of DAG above 5% in crude and interesterifiedburiti oil (*12*), favoring the formation of β′ polymorph. In addition, interesterification reduced TAG melting range, forming more unsaturated fatty acids and causing changes to crystal morphology and polymorphic forms. TAGs usually crystallize first in α and β′ forms even though β form is more stable. Factors such as cooling rate, crystallization heat, agitation level, and nanocarrier composition can affect the number and type of crystals. However, if fats are complex mixtures of TAGs, different polymorphic forms and liquid oil can coexist at a certain temperature (*47*).

**Table 3:** Short spacings (distance between parallel acyl groups in the triacylglycerol) and polymorphic (crystalline) forms of buriti oil, Compritol 888 ATO, NLC_BO_ and NLC_BO24h_ after 30 and 120 days of storage at 25 °C

Sample	*t*/day	Short spacing (Å)							Polymorphic form
4.6	4.4	4.3	4.2	3.9	3.8	3.7
Buriti oil			4.38^3^		4.19^3^	3.92^3^			α + β' + β
Compritol 888 ATO					4.25^4^		3.82^1^		β′
NLC_BO_	30	4.68^3^	4.45^3^		4.23^3^	3.95^3^			α + β′ + β
NLC_BO24h_	30	4.55^3^		4.32^3^	4.15^3^		3.88^3^		α + β′ + β
NLC_BO_	120	4.67^3^	4.46^3^		4.18^3^	3.93^3^		3.71^2^	α + β′ + β
NLC_BO24h_	120	4.63^3^	4.41^3^		4.19^3^		3.88^3^	3.68^2^	α + β′ + β

NLC_BO_=nanostructured lipid carrier prepared with non-interesterified buriti oil, NLC_BO24h_=nanostructured lipid carrier prepared with buriti oil interesterified for 24 h. ^1^weak, ^2^medium, ^3^strong, ^4^very strong

Solid lipids are predominantly found in the stable β-modification. When incorporated into lipid nanoparticles, the lipid transforms into the less ordered, metastable β′-modification, causing a distortion in the crystalline structure (*48*). Previous study demonstrated that the incorporation of lipids in NLCs leads to slower polymorphic transition and lower polydispersity index (*49*). In addition, NLC crystallization, melting temperatures and polymorphic content have been shown to be strongly dependent on the amount of oil. Jenning *et al.* (*50*) prepared NLCs with glyceryl behenate (Compritol 888 ATO) as the solid lipid and caprylic/capric triglycerides (Miglyol 812) as the liquid lipid. NLC dispersions were most stable in the absence of oil and at high concentrations of oil. Other studies showed that the addition of oil to solid lipid nanoparticles did not disrupt the structure of lipid crystals, contrary to the expected (*49*, *51*). Given the contradictory results, the effect of oil type on NLC polymorphic behaviour and morphology is still poorly understood (*21*).

#### Antioxidant activity of nanostructured lipid carriers

The ORAC assay measures the ability of an antioxidant to sequester free radicals through the donation of hydrogen atoms. Pulido *et al.* (*52*) described the FRAP method as an alternative to determine the ferric reducing capacity of biological fluids and aqueous solutions of pure compounds. These two assays are of great importance because they indicate the antioxidant action in foods and physiological systems.

Table 4 shows the results of the ORAC and FRAP assays. In both assays, NLC_BO24h_ had the highest antioxidant activity. NLC_BO24h_ had approx. 23% higher ORAC value and 16% higher FRAP value than NLC_BO_, demonstrating the influence of interesterification on the antioxidant activity of nanocarriers. Although there was a significant loss of tocopherols after 24 h of interesterification, the concentration of β-carotene was not affected and the concentrations of OOO-TAG (12.5%) and phenolic compounds increased, which could explain the increase in antioxidant activity (*12*). In addition, it can be considered that larger particles, such as NLC_BO_, tend to generate imperfect, amorphous crystals and can expel bioactive compounds, which causes a lower antioxidant capacity of these samples (*53*).

**Table 4:** ORAC value, linearity (area under curve *vs* concentration) and FRAP value of NLC_BO_ and NLC_BO24h_

Sample	ORAC assay					FRAP assay	
ORAC *c*(TE)/(μmol/mL)	*γ*(sample)/(mg/mL)	Linearity				FRAP *c*(TE)/(μmol/mL)
		Slope	Intercept	r^2^		
NLC_BO_	(1223.2±38.8)^a^	0.2–0.05	47.41	3.045	0.99		(939.6±53.0)^a^
NLC_BO24h_	(1592.4±94.9)^a^	0.2–0.05	53.52	3.489	0.98		(1116.1±11.5)^a^

ORAC=oxygen radical absorbance capacity, TE=trolox equivalents, NLC_BO_=nanostructured lipid carrier prepared with non-interesterified buriti oil, NLC_BO24h_=nanostructured lipid carrier prepared with buriti oil interesterified for 24 h. Results are expressed as mean value±S.D. (*N*=3). Values followed by different letters in superscript are significantly different at p<0.05

Haeiwa *et al.* (*54*) reported that unsaturated fatty acids, particularly oleic acid, increase the intracellular levels of lipid peroxidation products, indicating that oleic acid can promote adaptive response and protect cells against oxidative stress-related injury. Buriti oil contains considerable amounts of carotenoids, tocopherols, and phenolic compounds, which give it an important antioxidant power, favouring its own conservation and contributing to the treatment of oxidative stress-related diseases (*18*).

In a previous study carried out by our research group, the antioxidant capacity was also higher of the structured lipids produced with Lipozyme TL-IM and enzyme from *Rhizopus* sp., respectively, followed by pure buriti oil. This result indicates that enzymatic interesterification significantly increased the antioxidant capacity of the oil, independently of the content of minor compounds (*55*). In a study by Poyato *et al.* (*56*), the antioxidant capacity expressed as Trolox equivalents, measured by the L-ORAC (lipophilic radical absorption capacity of oxygen) assay, of linseed oil emulsions (8315.4 μmol/100 g) was higher than those of NLCs developed in the present study, whereas for olive oil emulsions (1978.9 μmol/100 g) was similar to those of NLCs. These data suggest that the antioxidant capacity of emulsions differs according to oil type and antioxidant compound.

The antioxidant activity of bioactive compounds is associated with the inhibition of free-radical chain initiation by oxygen binding or chelation of catalytic metal ions to retard oxidation and peroxide decomposition, prevent continuous hydrogen abstraction, and protect DNA, proteins and lipids against oxidative damage (*3*). The results of this study suggest that nanocarriers prepared with interesterified buriti oil can be used in several applications for the improvement of health, including cosmetic formulations, food products and pharmaceuticals.

## CONCLUSIONS

Interesterification reaction time significantly influenced particle size and interfacial properties of nanostructured lipid carriers (NLC). NLCs prepared with interesterified buriti oil formed more unsaturated TAGs and had smaller droplets than NLCs prepared with non-interesterified buriti oil. Although interesterification of buriti oil influenced the interfacial properties of droplets under the evaluated conditions, particles remained stable throughout the storage period. Besides that, NLCs showed a complex polymorphism with the presence of three crystalline forms and NLCs containing structured buriti oil had higher antioxidant capacity determined by ORAC and FRAP assays than NLCs without structured lipids. This research showed that interesterification positively influenced the physicochemical properties of NLCs, producing oils rich in oleic acid, with high stability and antioxidant capacity. Therefore, it may be interesting to use these nanocarriers to obtain efficient carrier systems for future applications.
